# Editorial: Water resource management using microbial solutions

**DOI:** 10.3389/fmicb.2026.1913841

**Published:** 2026-07-08

**Authors:** Bin Ji

**Affiliations:** Department of Water and Wastewater Engineering, School of Urban Construction, Wuhan University of Science and Technology, Wuhan, China

**Keywords:** agricultural wastewater, contaminant removal, groundwater contamination, microbial water treatment, sustainable water solutions, wastewater purification, water resource management

Water resource management is critically challenged by increasing demands and environmental limitations, particularly within agriculture, where both the purity and supply of water are essential for food security and ecosystem integrity. Microbial processes such as microbiologically induced precipitation and biodegradation represent a novel frontier in environmental biotechnology, offering sustainable pathways for treating and recycling water. For instance, innovations in energy- and resource-efficient biological nitrogen removal can mitigate reactive nitrogen pollution, microbial electrochemical systems and anaerobic processes purify wastewater while generating electricity from organic matter breakdown, and algae-based systems absorb excess nutrients, thereby improving overall water quality. Agricultural and industrial wastewater, often laden with complex mixtures of organic and inorganic pollutants, exemplifies the urgent need for such innovative treatments capable of addressing diverse contaminant profiles effectively. Concurrently, conventional wastewater treatment plants (WWTPs) are increasingly burdened by emerging contaminants, posing significant risks to both aquatic ecosystems and public health.

The Research Topic of this special issue, highlighting “*Water resource management using microbial solutions*,” comprises seven publications that offer in-depth insights into the latest developments spanning from mechanistic understanding of nitrogen removal to cutting-edge pathogen detection.

A foundational challenge in wastewater management is the efficient removal of nitrogen. In their study, Zhao et al. investigated short-cut nitrogen removal in a sequencing batch biofilm reactor treating ammonia-rich wastewater (1,000 mg-N/L). Their most distinctive contribution lies in elucidating the dual role of NO as both a toxic intermediate and a beneficial modulator that synergistically inhibits nitrite-oxidizing bacteria alongside free ammonia and free nitrous acid, thus facilitating the rapid establishment and long-term stability of partial nitrification. This mechanistic insight redefines NO from a mere process byproduct to an actionable control parameter. Complementing this reactor-scale perspective, Zhang M. et al. introduced a novel bacterial isolate, *Thauera* sp. JM12B12, which displays a dual-functionality of efficient aerobic denitrification coupled with the production of extracellular bio-flocculants. Remarkably, the strain achieved complete nitrate and nitrite removal at a low C/N ratio of 5, with total nitrogen removal exceeding 93%, while its cell-free supernatant demonstrated 91.4% flocculation efficiency. Genome analysis revealed a complete denitrification pathway including *napA*, two *nirS, norB, nosZ*, and 80 bio-flocculant-related genes on polysaccharide and protein production, making this strain a promising single-agent solution for simultaneous nitrogen and suspended solids removal for recirculating aquaculture systems.

Extending the nitrogen removal paradigm to groundwater remediation, Chen et al. explored the effects of coexisting Fe^2+^ and Mn^2+^ on sulfur autotrophic denitrification (SAD). Their key finding was that elevated Fe^2+^ concentrations significantly inhibited nitrate removal, reducing efficiency from 92.7 to 61.0%, accompanied by nitrite and N_2_O accumulation. This inhibition was mechanistically attributed to a decline in pH, a reduction in the relative abundance of the keystone sulfur-oxidizing genus *Thiobacillus*, and the downregulation of critical denitrification genes (*nirS, norB, nosZ*), and sulfur oxidation genes (*dsrA, soxB*). These results provide essential guidance for deploying SAD technology in iron- and manganese-rich waters, where metal ion interactions should be carefully managed.

While optimizing nutrient removal remains critical, the water sector faces an equally urgent challenge from emerging contaminants. Tsholo et al. conducted a comprehensive field survey of a South African WWTP and its receiving rivers, providing one of the first detailed assessments of antibiotic resistance gene (ARG) abundance and antimicrobial residues in sub-Saharan African surface waters. Their work revealed that elevated levels of multidrug-resistance genes persisted in river water downstream of the WWTP, with ampicillin concentrations posing a high ecological risk (RQ ≥ 1). The study further demonstrated significant correlations between specific bacterial genera and ARGs, implicating specific genera within *Proteobacteria, Bacteroidota*, and *Patescibacteria* as potential reservoirs of β-lactam resistance. These findings depict the urgent need to integrate ARG monitoring into routine water quality frameworks. In a parallel effort targeting recalcitrant industrial organics, Ahmed and Ray Chaudhuri developed a radio-tolerant bacterial consortium for the bioremediation of hexamine and formaldehyde. Their most notable achievement was demonstrating that the immobilized consortium maintained stable hexamine removal and regained full formaldehyde and COD removal capacity within 96 h following exposure to 5.25 Gray of ^60^Co γ-irradiation, with biofilm-based systems showing markedly faster recovery than suspended cultures. This represents a radio-tolerant microbial formulation for hexamine removal, opening avenues for treating complex effluents from nuclear medicine centers.

Underpinning all treatment and remediation efforts is the necessity for accurate and sensitive monitoring. Two studies in this Research Topic advance the frontier of pathogen detection. Daddy Gaoh et al. addressed the notorious difficulty of detecting fastidious specific microorganisms such as the *Burkholderia cepacia* complex in pharmaceutical-grade water. Their innovative approach combined oligotrophic enrichment using 1/10-strength TSB with shotgun metagenomic sequencing, which significantly improved the recovery and identification of BCC and other low-abundance pathogens compared to traditional rich media. This strategy highlights the power of tailoring culture conditions to the physiological niche of target organisms before molecular analysis. Pushing detection sensitivity to its practical limits, Zhang X. et al. developed a PMAxx-droplet digital PCR (ddPCR) assay for the absolute quantification of viable *Shigella flexneri* and *Shigella sonnei* in water. The key innovation lies in its duplex design targeting both a chromosomal marker and a virulence plasmid gene, enabling simultaneous species identification and pathogenicity assessment. With a detection limit of ≤ 10 CFU/reaction in fecal-spiked water samples, this assay provides a field-adaptable tool for water safety surveillance, though the authors appropriately caution that membrane integrity staining does not equate to cultivability and that plasmid loss may yield false negatives.

In conclusion, the studies presented in this Research Topic collectively illustrate a paradigm shift toward more mechanistically informed, multifunctional, and highly sensitive microbial technologies for water treatment and environmental health. From leveraging NO as a process-control agent and harnessing dual-function bacteria for integrated pollutant removal, to quantifying ARG dissemination in receiving waters and engineering radio-tolerant consortia for harsh environments, these contributions push the boundaries of what microbial systems can achieve. Complemented by advanced metagenomic and digital PCR tools that refine our ability to detect and quantify pathogens with unprecedented precision, this work lays a scientific foundation for next-generation water management. It is expected that this Research Topic will stimulate further interdisciplinary research, accelerating the translation of these promising microbial innovations into real-world solutions for a cleaner, safer, and more sustainable water future.

